# Successes of and Lessons From the First Joint eHealth Program of the Dutch University Hospitals: Evaluation Study

**DOI:** 10.2196/25170

**Published:** 2021-11-25

**Authors:** Anneloek Rauwerdink, Marise J Kasteleyn, Niels H Chavannes, Marlies P Schijven

**Affiliations:** 1 Department of Radiology and Nuclear Medicine Amsterdam UMC Amsterdam Netherlands; 2 Department of Public Health and Primary Care Leiden University Medical Centre Leiden Netherlands; 3 National eHealth Living Lab Leiden Netherlands; 4 Department of Surgery Amsterdam Gastroenterology and Metabolism Amsterdam UMC Amsterdam Netherlands; 5 Citrien Fund program eHealth Amsterdam Netherlands

**Keywords:** CSIRO framework, evaluation strategy, eHealth, telemedicine, qualitative research, formative evaluation, digital health

## Abstract

**Background:**

A total of 8 Dutch university hospitals are at the forefront of contributing meaningfully to a future-proof health care system. To stimulate nationwide collaboration and knowledge-sharing on the topic of evidence-based eHealth, the Dutch university hospitals joined forces from 2016 to 2019 with the first *Citrien Fund (CF) program eHealth*; 29 eHealth projects with various subjects and themes were selected, supported, and evaluated. To determine the accomplishment of the 10 *deliverables* for the *CF program eHealth* and to contribute to the theory and practice of formative evaluation of eHealth in general, a comprehensive evaluation was deemed essential.

**Objective:**

The first aim of this study is to evaluate whether the 10 deliverables of the *CF program eHealth* were accomplished. The second aim is to evaluate the progress of the 29 eHealth projects to determine the barriers to and facilitators of the development of the *CF program eHealth* projects*.*

**Methods:**

To achieve the first aim of this study, an evaluation study was carried out using an adapted version of the Commonwealth Scientific and Industrial Research Organization framework. A mixed methods study, consisting of a 2-part questionnaire and semistructured interviews, was conducted to analyze the second aim of the study.

**Results:**

The 10 deliverables of the *CF program eHealth* were successfully achieved. The program yielded 22 tangible eHealth solutions, and significant knowledge on the development and use of eHealth solutions. We have learned that the patient is enthusiastic about accessing and downloading their own medical data but the physicians are more cautious. It was not always possible to implement the Dutch set of standards for interoperability, owing to a lack of information technology (IT) capacities. In addition, more attention needed to be paid to patients with low eHealth skills, and education in such cases is important. The eHealth projects’ progress aspects such as *planning*, *IT services*, and *legal* played an important role in the success of the 29 projects. The in-depth interviews illustrated that a novel eHealth solution should fulfill a need, that partners already having the knowledge and means to accelerate development should be involved, that clear communication with IT developers and other stakeholders is crucial, and that having a dedicated project leader with sufficient time is of utmost importance for the success of a project.

**Conclusions:**

The 8 Dutch university hospitals were able to collaborate successfully and stimulate through a bottom-up approach, nationwide eHealth development and knowledge-sharing. In total, 22 tangible eHealth solutions were developed, and significant eHealth knowledge about their development and use was shared. The eHealth projects’ progress aspects such as *planning*, *IT services*, and *legal* played an important role in the successful progress of the projects and should therefore be closely monitored when developing novel eHealth solutions.

**International Registered Report Identifier (IRRID):**

RR2-10.1016/j.ceh.2020.12.002

## Introduction

### Background

The global population is increasing rapidly, and the number of people aged ≥60 years is expected to double by 2050 [1,2]. The direct consequences of global aging include rising health care expenditures and a potential shortage of health care professionals. The current COVID-19 pandemic has further uncovered the vulnerability of our health care systems and accessibility of, for instance, our hospitals in times of social distancing and a shortage of capacity [3,4].

Therefore, governments are increasingly debating which health care reforms are necessary to preserve the quality of our health care system and how to effectively deliver care from a distance. The concept of *eHealth* may support the necessary reforms [5]. By implementing eHealth solutions in daily practice, it is expected that health care processes would be executed more efficiently and subsequently time and costs would be saved [6-8]. Moreover, the use of eHealth can increase patient participation and empowerment [9,10]. In 2015, the World Health Organization Global Observatory for eHealth explored eHealth developments and investigated how eHealth can support universal health coverage [11]. The report considered eHealth foundation built through policy development, funding approaches, and training of students and professionals. Policy development is of utmost importance to counteract the *fragmentation* of eHealth initiation [12]. In addition to policy development, it is important to investigate how to develop and implement novel eHealth solutions at scale successfully. Schreiweis et al [13] summarized the critical factors influencing the implementation and adoption of eHealth. They described the perceived barriers, such as added workload, problems with financing, and missing fit in organizational structures. In addition, a lack of system interoperability is a well-known and frustrating issue in preventing the sustainable implementation of eHealth [14]. Finally, it seems that developers, evaluators, and physicians find it difficult to learn from successful initiatives that come from external sources, such as those from other disciplines or from outside the region [15]. They have the so-called “not-invented-here syndrome.”

In the Netherlands, as part of the Dutch national eHealth strategy, the *Citrien Fund* (CF) was established for the period of 2014 to 2018 by the government-funded Netherlands Organisation for Health Research and Development (in Dutch: ZonMw). The CF aims to contribute to a sustainable health care system by stimulating collaboration between 8 Dutch university hospitals, and between the university hospitals and other health care organizations. The CF supports 5 programs with different themes [16]. This study focuses on the *CF program eHealth,* which took place from 2016 until the beginning of 2019. This first nationwide university hospital eHealth collaboration mainly focused on the constitution of a strong collaborative framework to discuss present eHealth issues, on the development of a wide array of novel eHealth solutions—with the most successful being scaled in a subsequent program—and on sharing of eHealth knowledge. The overall aim of the *CF program eHealth* was to accomplish 11 predefined deliverables (Textbox 1), which were drafted upon knowledge gaps within the Dutch eHealth landscape. To achieve deliverables 2 to 11, 29 eHealth research projects were conducted.

### Objectives

By conducting a comprehensive evaluation of the *CF program eHealth*, including determination of the barriers to and facilitators of the development of each of the 29 projects, this study aims to assess the successes of and lessons from the *CF program eHealth*. This study also aims to reduce the scarcity of formative eHealth evaluations and to become a useful case study for the eHealth evaluators of eHealth development programs [17-19]. Subsequently, the successful future development and implementation of eHealth, in general, might be enhanced. This study consists of 2 aims: (1) to evaluate whether the 10 deliverables of *CF program eHealth* were accomplished and (2) to evaluate the progress of the 29 eHealth projects to determine the barriers to and facilitators of the development of the *CF program eHealth* projects.

**Deliverables of the *Citrien Fund program eHealth* [20].**

1. One coordinating Dutch Federation of University Medical Centers vision on eHealth and eHealth road map (the accomplishment of this deliverable was reported in a previous study and is, therefore, not part of this study [21]).
2. A virtual nationwide expertise center for eHealth.
3. International positioning by promoting in journals and media and during the closure event.
4. Conditions for downloading medical data described and, if possible, realized.
5. Blueprint for interoperability between hospital information systems and electronic health records.
6. Agreements and standards for data sharing between consumer and professional eHealth.
7. A framework for regional collaboration for effective implementation of eHealth.
8. Models that can strengthen the empowerment of the patient.
9. A developed multidisciplinary infrastructure to stimulate the development of digital health.
10. Development, evaluation, and implementation of eHealth instruments in collaboration with companies and start-ups.
11. A blueprint for education in eHealth competencies and skills for health care professionals.

## Methods

### Setting

As described in the previous study protocol, each of the 8 Dutch university hospitals was asked to propose 3 or 4 eHealth research projects to be carried out in their own university hospital. Although the university hospitals had a carte blanche for the projects they proposed, every project had to contribute to one or more of the 10 program deliverables. In total, 29 projects covering a wide array of eHealth themes were carried out from June 2016 to January 2019 [16]. The projects delivered either a tangible eHealth solution or provided knowledge about the use or development of an eHealth solution. Each project was managed by a dedicated local project leader, with 29 project leaders in total. At the initiation of the *CF program eHealth,* each project leader drafted a detailed project plan describing which of the 10 program deliverables the project aimed to contribute, the project’s objectives, and a detailed timeline. The project plan was approved by the supervising steering committee, which consisted of 2 representatives of each university hospital. In a 3-month meeting, the steering committee monitored the progress of the projects and advised the project leaders. In addition, at the midterm (December 2017) and end-term (October 2018) of the program, the project leaders presented their projects’ progress to the steering committee, and a majority decision regarding the continuation of the project was made.

### Evaluation Study

**Figure 1:**
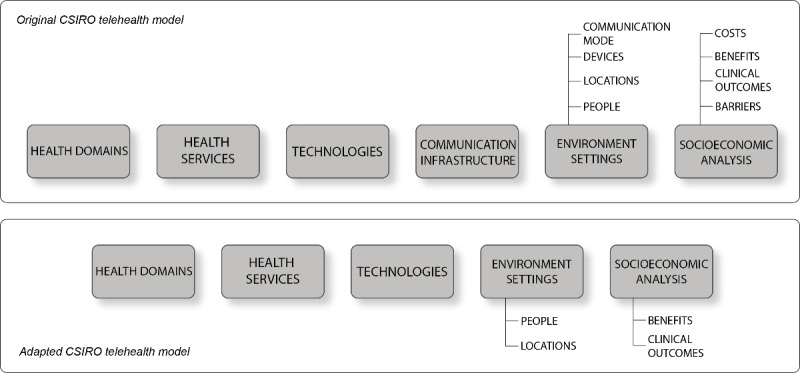
The original Commonwealth Scientific and Industrial Research Organization (CSIRO) model versus the adapted CSIRO telehealth model.

An adapted version of the *Commonwealth Scientific and Industrial Research Organization (CSIRO) framework for evaluating telehealth trials or programs* (Figure 1) was deemed the most suitable framework for the evaluation of the *CF program eHealth* (Textbox 1), as described in a previous study protocol paper [16,22]. The following aspects of the adapted CSIRO framework for each project were described: health domain, health service, technology, environment setting, and (clinical) outcomes or evident benefits. Owing to the wide array of projects, covering various topics and using diverse study designs, the last aspect was broadly defined, ranging from usability outcomes to clinical outcomes, and qualitative eHealth insights. The adapted CSIRO framework was incorporated into the *Citrien Fund – mapping table* (Multimedia Appendix 1), which also presents general information and the completion status for each project’s deliverables.

In the *program deliverables* column of the mapping table, the deliverables that the project aimed to achieve were indicated with red, orange, or green dots depending on the level of accomplishment at end-term. The red dot indicated that the project failed to accomplish the deliverable, orange indicated partial completion, and green indicated full accomplishment of the deliverable. Based on this overview, a short summary for the completion of each deliverable is provided.

During the end-term presentation, the project leaders presented the project’s main findings to the steering committee. This presentation included all aspects of the adapted CSIRO framework and was used to systematically collect the data for each project.

### Mixed Methods Study

**Figure 2:**
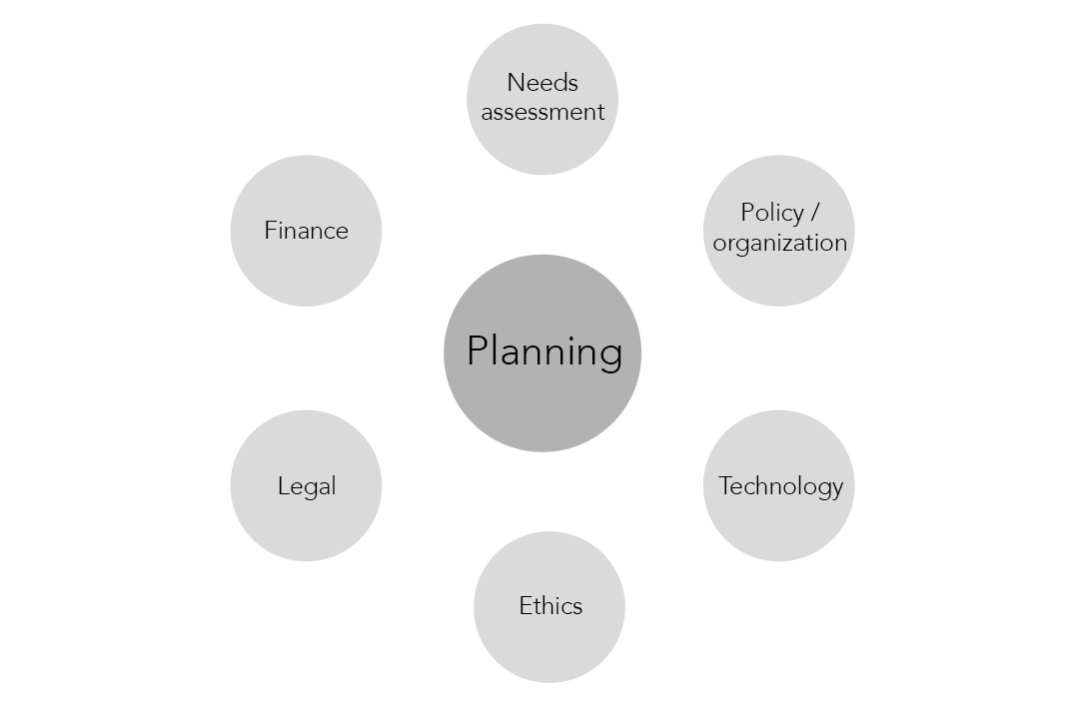
eHealth project progress aspects.

Through a mixed methods approach, consisting of a questionnaire and a semistructured interview, the barriers to and facilitators of the development of the 29 eHealth projects *eHealth* were determined. Previously, it had been found that 7 eHealth project progress aspects were useful for monitoring the progress of eHealth projects: planning, needs assessment, policy or organization, technology, ethics, legal, and finance (Figure 2) [16]. By quantitatively and qualitatively evaluating these 7 aspects for the 29 projects, we aimed to obtain insights into which barriers and facilitators were important for the successful development of the *CF program eHealth* projects.

#### Questionnaire

All project leaders were asked to fill out a self-developed 2-part questionnaire. The main concepts that the questionnaire covered were the eHealth project progress aspects: planning, policy or organization, technology, ethics, legal, and finance. Various question formats were used, including yes or no, multiple-choice, and 4-point Likert scale questions. Part one had to be completed at the midterm (52 questions) and part two at the end-term (55 questions) for each project. With this longitudinal aspect, we aimed to evaluate the change in magnitude of the eHealth project progress over time. The topics included respondent demographics and items related to eHealth project progress aspects. The questionnaire was completed using the web-based survey software SurveyMonkey (SurveyMonkey, Inc) [23]. The respondents received an email with an invitation link to the questionnaire and reminder emails were sent to them at 3- and 5-week intervals.

#### Interview

At the end of the *CF program eHealth,* one project leader from each university hospital was randomly selected by drawing lots and interviewed by the coordinating researcher (AR) for a more in-depth exploration of the role of the eHealth project progress aspects in their project. The semistructured interviews were held by telephone, following a previously composed interview guide (Multimedia Appendix 2), which had a subset of open questions on each eHealth project progress aspect and used an iterative approach. The same set of questions was used in all interviews and, if necessary, adjusted along the way. The interview guide was composed of inputs from the results of the questionnaire and notes from the 3 monthly meetings of the steering committee. It was estimated that saturation was reached after 8 interviews; however, if saturation had not been reached, more project leaders were interviewed.

### Data Analysis

The quantitative questionnaire data were analyzed by calculating descriptive statistics using Microsoft Excel. Continuous data were summarized using median and IQR. Categorical data were presented as frequency counts with percentages.

The 8 interviews were digitally recorded with the permission of the project leaders and transcribed verbatim. AR analyzed the transcripts according to the 6-step thematic analysis framework of Braun and Clarke [24] and discussed the results with the last author (MPS). First, we read the transcripts thoroughly to familiarize ourselves with the data. Second, notes were placed next to the text to generate the initial codes. Finally, major themes were identified and defined, and the data were further coded and sorted into themes and subthemes. The data from each theme were summarized into descriptions concerning the contribution of the 7 eHealth project progress aspects in the successful performance of an eHealth project.

## Results

The results of the evaluation study are schematically presented in the *Citrien Fund - mapping table* (Multimedia Appendix 3). In total, 22 projects developed a tangible eHealth solution, and 7 projects acquired knowledge about the development and use of eHealth solutions. The projects were conducted in various *health domains*, of which internal medicine represented the biggest share. In the *health services* column table, it is shown that most of the projects contributed to nonclinical services, such as education, administration, and research. Projects contributing to clinical services, such as treatment, monitoring, and diagnostics, were mentioned in a minority of cases. In the *environment setting*, 17 projects focused on patients, 14 on health care providers or physicians and 6 on researchers. A total of 14 projects were related to a home-based environment and 6 to a hospital environment.

### Evaluation Study

All 10 deliverables of the *CF program eHealth* were successfully achieved. The contributions of the 29 projects to the 10 deliverables are summarized in Table 1. In summary, most of the projects (19/29, 66%) contributed to models that strengthen the patients’ directing role (deliverable 8), making *strengthening the patients’ directing role* the most prominent theme of the *CF program eHealth*. We learned that the patients were enthusiastic about accessing and downloading their own medical data on the web (deliverable 4), but physicians were more cautious, and it was not always possible to implement the Dutch set of standards for interoperability owing to a lack of information technology (IT) capacities (deliverable 5). We also learned that considerable attention should be paid to eHealth literacy when developing novel eHealth solutions (deliverable 4). Furthermore, the establishment of alternative communication infrastructures (deliverable 9) between the caregiver within the hospital and the patient outside the hospital was investigated and considered very relevant.

**Table 1:** Summary of major findings per deliverable.

Deliverable	Projects^a^	Conclusion
2	24	A Dutch nationwide web-based expertise center for eHealth was established, containing an eHealth toolkit with the various delivered products and the acquired knowledge [25].
3	4, 13, 15, 16, 20, and 21	Various projects published their results in national and international (scientific) journals [26-33]. In addition, an e-book has been published [34].
4	5, 9, 10, 13, 19, 23, 24, and 26	In general, the patients were enthusiastic about accessing their medical data on the web and downloading them. Physicians were still holding back. eHealth literacy must be considered in the development and implementation of eHealth. One of the university hospitals dealt with information exchange between the primary care and the hospital, for which a legal framework had been set.
5	5, 13, 19, 23, and 25	It was not always possible to implement the Dutch set of standards for interoperability. Integrating different IT^b^ systems for exchanging data was complex and might not be desirable in a pilot phase.
6	14, 19, 23, 25, and 26	An important insight obtained was that when exchanging data, the skills of the end user should be considered. Attention should also be paid to patients with low eHealth skills.
7	5, 16, 18, 23, 24, and 29	Within the projects, there was frequent co-operation with IT developers in the region and the first- and second-line health care institutions.
8	1, 4, 5, 6, 7, 10, 13, 14, 15, 16, 17, 18, 22, 24, 25, 26, 27, 28, and 29	Most of the projects (19/29, 66%) contributed to models that strengthen the patients’ directing role. For example, 2 e-learnings were developed, in which both the patient and the caregiver received the tools to make better decisions together. In addition, several mobile apps or web-based applications that were developed, for example a medical dashboard, a patient coach, an app for glycemic index, a web-based blog and forum for patients with Alzheimer, and a home-based blood pressure monitor for high-risk pregnant women, reinforced the patients’ directing role.
9	15, 16, 18, 19, and 28	Several projects focused on establishing alternative communication infrastructures between a hospital and a patient outside the hospital. In addition, for the development of these new type of eHealth solutions, the projects required close co-operation and consultation with researchers, patients, informal caregivers, IT services, and lawyers.
10	1, 2, 3, 4, 6, 7, 8, 11, 13, 14, 15, 16, 17, 20, 21, 24, 26, 27, 28, and 29	A website with a forum and a blog was developed for patients with Alzheimer. Also, a total of 8 mobile apps were developed. Various wearables were tested for the home monitoring of patients. A clinical data science eBook was made, and several e-learnings were developed. The efficacy and effectiveness of the various eHealth solutions were scientifically evaluated.
11	3, 8, 12, 14, 15, 17, 21, 24, and 27	Several projects contributed to improving eHealth education. For example, the *develop your own eHealth app* project developed an education module in which medical students learned about eHealth and the necessary eHealth skills. Another project translated the English language Apple Research Kit into a Dutch variant and offered researchers a guide on how to conduct eHealth research with the kit. Finally, some projects also focused on patient education and how to enable patients with low health skills to work with eHealth tools.

^a^The project numbers correspond with the projects illustrated in the Citrien Fund - mapping table in Multimedia Appendix 3.

^b^IT: information technology.

Owing to insufficient progress, one project was prematurely terminated by the steering committee after the midterm evaluation, and another 3 projects were prematurely terminated after the end-term evaluation. These projects are indicated with an asterisk (*) in the *Citrien Fund - mapping table*. As shown in the *(Clinical) outcomes or evident benefits* column, in 2 of the 4 cases, personnel matters were responsible for the insufficient progress, in one case there was an issue with the intraoperability of the proposed eHealth solution with the existing IT system and in the last case there was an *IT freeze* hospital-wide because of the implementation of a new electronic health record system.

### Mixed Methods Study

#### Questionnaire

The 2-part questionnaire was completed by all the participating project leaders in November 2017 and October 2018. The first part was completed by 29 project leaders, and the second part was completed by 27 project leaders owing to the premature termination of 1 project and the early completion of another. Tables 2 and 3 show the main characteristics of the participants and the projects, respectively.

**Table 2:** Demographics of project leaders (N=29)^a^.

Demographics			Values, n (%)
**Gender**			
	Female	17 (59)	
	Male	12 (41)	
**Degree^b^**			
	Medicine	7 (19)	
	Psychology	4 (11)	
	Health sciences	3 (8)	
	(Medical) biology	2 (6)	
	Communication	2 (6)	
	Other^c^	18 (50)	
**Employment**			
	Hospital	22 (76)	
	Outside hospital	3 (10)	
	Both	4 (14)	
**Weekly time spent at the project (hours)**			
	<5	9 (31)	
	5-10	12 (41)	
	10-15	3 (10)	
	>15	5 (17)	
Age (years), median (IQR)			33 (29-44.5)

^a^Measured midterm.

^b^More degrees per project leader possible.

^c^One degree per other specialty.

**Table 3:** General characteristics of projects (N=29)^a^.

Characteristics			Values
**Reasons for participating in the *CF*^b^ *program eHealth, n (%)***			
	Subsidy	25 (86)	
	Publicity	19 (66)	
	Collaboration	20 (69)	
	Other reasons	5 (17)	
**If the project was not accepted in *CF*^b^ *program eHealth*, then, n (%)**			
	It would have remained a project plan	8 (28)	
	I would have actively searched for other means	21 (72)	
	Other existing means were allocated to the project	0 (0)	
**Number of people involved internally, n (%)**			
	0	1 (3)	
	1-2	3 (10)	
	3-4	15 (52)	
	>5	10 (34)	
**Number of people involved externally, n (%)**			
	0	6 (21)	
	1-2	11 (38)	
	3-4	6 (21)	
	>5	6 (21)	
**Number of monthly meetings with steering committee member, n (%)**			
	0-1	13 (45)	
	1-2	7 (24)	
	>2	4 (14)	
	Never	5 (17)	
**Time to acceptation in months, median (IQR)**			0 (0-4)

^a^Measured midterm.

^b^CF: Citrien Fund.

The main reason for participating in the *CF program eHealth* was to receive funding (25/29, 86%). However, publicity (19/29, 66%) and collaboration (20/29, 69%) were also important reasons. Of the 29 projects, 25 (86%) had ≥3 people internally involved, and in 23 (79%) projects, people from outside the hospital, such as general practitioners, patients, and software developers, were involved as well.

The main results of the questions concerning *planning* have been presented in Table 4. At midterm, all the project leaders (29/29, 100%) estimated that they would be able to complete the selected program deliverables, as described in their project plan. However, at end-term, 4 project leaders (4/27, 15%) indicated that they might not be able to contribute to the deliverables. At midterm, almost half (12/29, 41%) of the project leaders indicated that their project planning was no longer up to date. Moreover, at the end of the study, the planning of the majority (23/27, 85%) was not up to date. In addition, at midterm, 6 project leaders (6/29, 21%) indicated that the time available for successful progress in the project was not sufficient; this share doubled (12/27, 44%) at the end of the questionnaire.

**Table 4:** eHealth project progress aspect *planning*.

Question	Midterm (n=29)		End-term (n=27)	
	Yes, n (%)	No, n (%)	Yes, n (%)	No, n (%)
Is the available time sufficient for successful progress of your project?	23 (79)	6 (21)	15 (56)	12 (44)
Is your project planning, as described in the project plan, still up to date?	17 (59)	12 (41)	4 (15)	23 (85)
As the project is progressing now, I expect to achieve the measurable goals as described in the project plan	25 (86)	4 (14)	19 (70)	8 (30)
I do not foresee any problems in contributing to program deliverables at the end of the Citrien Fund program eHealth, as described in my project plan	29 (100)	0 (0)	23 (85)	4 (15)

Table 5 presents results regarding the eHealth project progress aspects, *policy* or *organization*, *technology*, *ethics*, and *legal*. None of the topics was spared of inconvenience, with IT services and privacy issues representing the greatest shares in moderate to significant inconvenience.

Regarding the aspect of *finance*, 8 projects (8/29, 28%) received additional funding other than that from the *CF program eHealth* at the initiation of the project. Furthermore, at midterm, 8 project leaders (8/29, 28%) thought that they would need extra funding for the successful completion of their project. However, at the end of the study, 13 project leaders (13/27, 48%) stated that extra funding would be necessary for project completion. The funding received from the *CF program eHealth* was mostly insufficient to cover the personnel expenses and the implementation aims. After termination of the *CF program eHealth*, of the 27 projects, 13 (48%) still needed to find financial means to continue, while 4 (15%) already had the means and 10 (37%) did not need any.

**Table 5:** Inconvenience issues encountered during project execution (N=27).

	No inconvenience, n (%)	Some inconvenience, n (%)	Moderate inconvenience, n (%)	Significant inconvenience, n (%)	Not applicable, n (%)
Realizing contracts with third parties	7 (25)	6 (22)	7 (25)	3 (11)	4 (14)
Privacy issues, such as patient data protection	9 (33)	4 (14)	7 (25)	5 (18)	2 (7)
Review of medical ethics committee	7 (25)	8 (29)	5 (18)	4 (14)	3 (11)
Resistance from within the organization	8 (29)	9 (33)	8 (29)	2 (7)	0 (0)
Resistance from outside the organization	13 (48)	10 (37)	4 (14)	0 (0)	0 (0)
Information technology developers and support	7 (25)	5 (18)	6 (22)	6 (22)	3 (11)
Electronic health record supplier	8 (29)	3 (11)	0 (0)	3 (11)	13 (48)

#### Interview

In total, 8 project leaders were interviewed, representing the entire group of project leaders. After 7 interviews, saturation of information was reached. Three major themes, with subthemes, were identified (Textbox 2). The main findings of the interviews have been discussed under these 3 themes, supported by quotes (Multimedia Appendix 4) as examples of the participants’ responses.

**Themes and subthemes identified from interviews.**

**Success factors for eHealth development and implementation**

- Fulfill a need
- Outsource
- Communication
- Personnel

**Essential third parties**

- Information technology services
- Medical Ethical Committee
- Legislation

**Flexibility**

- Project planning
- Conducting research
- Effectiveness testing

#### Theme: Success Factors for eHealth Development and Implementation

While carrying out their eHealth projects, the project leaders encountered several relevant aspects that contributed to the success of their projects. In such cases, there should be an evident *need to fulfill*, or problem to solve, when developing an eHealth solution, although conducting a needs assessment was not deemed necessary. Regarding *outsourcing*, it was crucial to find the right (commercial) partners that already had the knowledge and means to accelerate development. Clear *communication* with IT developers and other stakeholders about the development and other concerns, for instance, estimated changes in routine care, was essential. Successful development of an eHealth solution may depend on the availability and dedication of the *personnel*, including the project leader.

#### Theme: Essential Third Parties

Owing to the immaturity and complexity of eHealth, several important topics related to its development and implementation have never been discussed before. Therefore, communication and close collaboration with third parties was of utmost importance.

Regarding *IT services*, the project leaders indicated that communication was the number one pitfall in successful collaboration. It should also be emphasized that IT development costs time, capacity, and money. *Medical ethical committees* may find it difficult to take a position because of the unknown impact or burden of a novel eHealth solution. Moreover, one should be informed about the impact of *legislation* on their project. In addition, a privacy impact assessment is often obligatory, which may cause a delay in planning.

#### Theme: Flexibility

eHealth solutions are considered complex interventions with multiple interacting components. This required some level of flexibility when it came down to project planning, conducting research, and effectiveness testing. *Project planning* is important in the initial stages. However, it should be possible in case of incidents to make timely adjustments.

Regarding *conducting research,* there were varying responses to whether it was challenging to find study participants to evaluate the eHealth project. However, it seems that when an eHealth solution can solve a relevant problem, patients are willing to participate. Furthermore, the study end point was difficult to determine because of the novel character of eHealth and the resulting lack of literature. eHealth solutions should be evaluated in a study context where possible. Nevertheless, it was considered unnecessary to conduct a randomized controlled trial to prove *effectiveness*, because, for example, patients had already experienced significant benefits while using a novel eHealth solution. In addition, while clinical effectiveness may be obligatory, it does not tell anything about the effectiveness of the eHealth solution.

## Discussion

### Principal Findings

#### Evaluation Study

The evaluation study targeted the first study aim. By systematically evaluating the accomplishment of 10 program deliverables, we were able to determine the successes and lessons of the *CF program eHealth*. In this study, we learned that patients are more enthusiastic about downloading their own medical data than physicians, that the lack of IT capacities plays a negative role in implementing the Dutch set of standards for interoperability, that considerable attention should be paid to patients with lower eHealth skills, and that establishing alternative communication infrastructures between caregivers and patients should be considered very important.

The 29 different eHealth research projects delivered 22 tangible eHealth solutions and significant knowledge about the development and use of eHealth solutions. Strengthening the patient’s directing role was the most prominent theme of the *CF program eHealth.*

Through the formation of a new collaborative network, the *CF program eHealth* was able to bring the 8 Dutch university hospitals together and therewith significantly improve the fragmented eHealth landscape in the Netherlands.

#### Mixed Methods Study

The mixed methods study targeted the second study aim. The questionnaire helped us learn that the eHealth project progress aspects *planning*, *technology*, and *legal* played an important role in successful development of the 29 projects. However, a lack of time with the individual project leaders, priorities other than implementing eHealth in IT services, and the never discussed before privacy issues, together with the relatively short *CF program eHealth* duration, caused project delays.

In the in-depth interviews, the themes: *success factors for eHealth development and implementation*, *essential third parties*, and *flexibility*, were identified as the 3 most important themes to pay attention to when carrying out an eHealth project.

#### National eHealth Programs

Although it seemed that the Netherlands was among the first countries to carry out a publicly funded university hospital eHealth program, there are other nationwide eHealth programs and initiatives with which the *CF program eHealth* could be compared.

The National Health Service (NHS), the publicly funded health care system in England, holds an active and well-organized digital section, the *NHS digital* [35]. One of its programs encompasses the initiation of a physical and conceptual digital, research, informatics, and virtual environments (DRIVE) unit, which explores, among other topics, how to gain insights from machine learning and artificial intelligence (AI). To the best of our knowledge, the program is not scientifically evaluated nor is it visible to the public what has been done. In addition, the NHS only collaborates with the Great Ormond Street Hospital, whereas the collaborative network of the *CF program eHealth* encompasses all university hospitals of the Netherlands. Furthermore, as described by Astana et al [12], the top-down approach of the NHS and fragmentation with respect to the organization and delivery of care, makes it a complex landscape for eHealth companies seeking to enter the system and scale up innovation [12]. In contrast, the *CF program eHealth* used a bottom-up approach to search for innovative initiatives, controlled by the 8 university hospitals rather than the government itself. Owing to the high visibility of this program on the web and the co-operation with stakeholders and patients, many useful insights into nationwide eHealth development were gathered and disseminated directly.

The Danish program *Patient@home* focused on rehabilitation and monitoring services to promote patient empowerment and support treatment at home [36]. In total, 30 projects were carried out through public-private collaborations between patients, research institutions, and other stakeholders. Although all the projects were developed using structured 5-phase innovation models, with the backgrounds and aims as described in detail on the program’s website, no scientific results were reported on the website [37]. In the case of the *CF program eHealth*, projects may have benefited from a structured innovation model with subsequent phases of development and implementation. Especially in the case of the 4 projects that were prematurely terminated, an evaluation of the first phase of innovation—*need*—which includes technology screening, could have been beneficial. However, the well-thought program structure, consisting of an obligatory project plan and a planning stage at the initiation of a project and 2 project progress evaluations along the way, might have overcome the lack of a structured innovation model.

#### eHealth Project Progress Aspects

The mixed methods study found that the eHealth project progress aspects *planning*, *technology*, and *legal* were important aspects in relation to successful progress of the projects. From an implementation perspective, comparable results were described by Schreiweis et al [13]. After conducting a systematic literature and expert discussion analysis, the authors considered flexible funding, health outcomes, policies for using generated data for research, competition, and supporting laws and regulations, as important factors for success. Our study added insights into the successful project progress from a developmental perspective. For example, having a dedicated project leader was essential, as were flexible project planning, clear communication with IT services, and collaboration with (commercial) partners that already had the knowledge and means to accelerate development. Liu et al [38] studied the barriers to and facilitators of such an academia-industry collaboration. They identified the aspect timeline, consisting of longer time frames in research projects, contrasting with the greater emphasis on quick implementation in industry, as a barrier to successful academia-industry collaboration. To mitigate this, our study found that outlining and communicating openly about the goals and expectations may facilitate successful academia-industry collaborations.

Vedlūga and Mikulskienė [39] compiled a corpus of indicators to monitor the implementation of the national eHealth information system and proposed 5 key dimensions of stakeholder-driven performance elements for eHealth evaluation: human resources, financial resources, management resources, legal aspects, satisfaction with technological solutions, and design. These performance elements match with the results of our study, such as the issues caused by misunderstandings between clients and IT service providers, shortage of funding, and never discussed before legal issues. The element human resources was considered less important to eHealth development. However, close attention should be paid to the endemic problem of researchers performing work in their spare time.

### Strengths and Limitations

One of the major strengths of the *CF program eHealth* was the open and collaborative organization with a bottom-up approach. For example, one representative from each of the 8 Dutch university hospitals took place in the steering committee and was responsible for monitoring and mentoring local projects. A solid foundation was laid for successful program progress and the achievement of program deliverables.

Another strength was the wide array of subjects and studies in the 29 eHealth projects. Although this variation may have reduced comparison possibilities and thus the external validity of projects, many valuable insights that proved important across settings were gained. However, by creating a systematic overview of the projects’ findings in the evaluation study, their comparability was greatly enhanced. The combination of an evaluation study with a mixed methods study to evaluate the progress of eHealth projects in detail further strengthens our study findings.

A study limitation was that the *CF program eHealth* deliverables were not formulated through the well-known Specific, Measurable, Attainable, Relevant, and Timely formats. Therefore, it might be debatable whether the 10 deliverables were truly completed. Another limitation regarding the achievement of the deliverables might be the finding that most of the project leaders indicated in the questionnaire that their project planning was no longer up to date at the end of the *CF program eHealth*. However, a *not anymore up to date* project plan did not necessarily mean project failure and, therefore, its failure to achieve deliverables. Project planning was relevant to the initial direction and evaluation progress but on-time completion of project planning was not compulsory when drawing the final conclusions about the results of a project.

A limitation regarding the mixed methods study might have been the number of interviewed project leaders. However, saturation of information was accomplished after 7 interviews, and the interviewed project leaders were a representative sample of the whole group.

A final limitation was that we used a self-developed questionnaire to assess the eHealth project progress aspects of the 29 projects. We decided to develop a questionnaire owing to the relatively small group of participants and the limited time frame. Although valuable insights were gained, methodologically, it would have been stronger if some level of content validity and construct validity was carried out.

### Future Perspectives

After termination of the *CF program eHealth,* the most successful projects have now scaled up nationwide in a subsequent edition of the *CF program eHealth,* focusing on implantation and upscaling [40]. The lack of sequential funding, as indicated by many in the Mixed Methods Study section of this paper, will be overcome for these projects. In addition, the assumption that most eHealth projects suffer from *pilotitis* (ie, projects will never pass the pilot phase), might also be overcome with the sequel of *CF program eHealth* [17].

The COVID-19 pandemic has accelerated the uptake and implementation of existing eHealth solutions [41,42]. Although care delivered at a distance has been already possible for many years, actual scaled-up use is still lacking. The aforementioned aspects of technology and law, which slowed the progress of the development of the projects, might be positively influenced and could take eHealth to a more mature level in the aftermath of this crisis. However, new points of discussion will need to be taken care of, such as how to take care of data privacy and legislation if a nationwide COVID-19 mobile app was implemented [43].

The *CF program eHealth* has proven that nationwide collaboration on the theme of eHealth between the 8 Dutch university hospitals and related commercial parties is possible and diminishes eHealth fragmentation. To truly preserve and improve the quality of our health care system in the light of global aging, we should strive for the elimination of eHealth fragmentation and national and international eHealth collaborations, such as the *CF program eHealth,* should be stimulated by governments and the European Union.

### Conclusions

The 8 Dutch university hospitals were able to successfully collaborate and stimulate nationwide evidence-based eHealth development using a bottom-up approach. In total, 22 novel eHealth solutions with various subjects were developed, and significant knowledge about eHealth development and use was established. The aspects *planning*, *technology*, and *legal* played an important role in successful progress of the projects and should therefore be closely monitored while developing novel eHealth solutions or when implementing existing solutions. To further counteract eHealth fragmentation and take the next step from development to upscaling, a subsequent *CF program eHealth* will be carried out.

## Appendix

Multimedia Appendix 1 Empty Citrien Fund - mapping table.

Multimedia Appendix 2 Interview guide.

Multimedia Appendix 3 Citrien Fund - mapping table.

Multimedia Appendix 4 Interview quotes.

## Abbreviations

AI: artificial intelligence
CF: Citrien Fund
CSIRO: Commonwealth Scientific and Industrial Research Organization
DRIVE: digital, research, informatics, and virtual environments
IT: information technology
NHS: National Health Service
