# Supplementary material for: Successes of and Lessons From the First Joint eHealth Program of the Dutch University Hospitals: Evaluation Study
Source: J Med Internet Res. 2021 Nov 25;23(11):e25170. doi: 10.2196/25170 (PMC8663485; doi:10.2196/25170)
Supplement: Multimedia Appendix 2 [file jmir_v23i11e25170_app2.docx]

|  |  |
| --- | --- |
| Topic list | Probing questions |
| Project management / planning | Did you perform a needs assessment at the start of the project? Why yes or no? |
|  | Was it difficult to draft the obligated project plan with the SMART goals? |
|  | How did your project planning proceed? Described factors that influenced your planning. |
|  | To which extent is a strict planning important for success of an eHealth project? |
|  | Did you depend on, internal or external, third parties? How did the influence the progress? |
| Policy / organization | To which extent could your project change a process within a health care organization? |
|  | Did you encounter resistance from your organisation while carrying out your project? How? |
| Technology | Did you encounter resistance from IT services, e.g. developers or EHR providers? How? |
|  | To which extent is IT important for success of an eHealth project? |
|  | Would it be difficult to keep the technology up to date? |
|  | Did you test usability? What happened? |
| Ethics | Did your project need approval from a medical ethics committee? How did that go? |
|  | Did you encounter resistance within this topic while carrying out your project? How? |
|  | To which extent are ethics important for success of an eHealth project? |
| Legal | Did your project include legal topics? Such as? |
|  | Was there a data protection officer involved? |
|  | Did you encounter resistance within this topic, e.g. privacy and security issues, while carrying out your project? How? |
| Finance | Did you describe a budget at the start of the project? Why yes or no? |
|  | What do you think about the amount of funding? Was it sufficient? |
|  | After termination of the program, how will the project be financed? |
| Other | Are there other aspects that influenced the progress of your project? |
|  | In general, what makes an eHealth project successful? |
|  | What is your opinion about 'evidence-based eHealth'? Do you think it is necessary to investigate eHealth in a study context? |

Multimedia Appendix 3. Interview guide
