# Supplementary material for: Successes of and Lessons From the First Joint eHealth Program of the Dutch University Hospitals: Evaluation Study
Source: J Med Internet Res. 2021 Nov 25;23(11):e25170. doi: 10.2196/25170 (PMC8663485; doi:10.2196/25170)
Supplement: Multimedia Appendix 3 [file jmir_v23i11e25170_app3.docx]

Multimedia Appendix 3. Citrien Fund - mapping table

| **Project title** | **Theme** | **Project type** | **Deliverables&** | | | | | | | | | | **CSIRO framework** | | | | |
| --- | --- | --- | --- | --- | --- | --- | --- | --- | --- | --- | --- | --- | --- | --- | --- | --- | --- |
| *AMC* | | | *2* | *3* | *4* | *5* | *6* | *7* | *8* | *9* | *10* | *11* | *Health domain* | *Health service* | *Technology%* | *Environment setting#* | *(Clinical) outcomes or evident benefits$* |
| E-learnings for shared decision-making | Digital interaction | Product |  |  |  |  |  |  | • |  | • |  | NA | Education, training | eLearning | Patients and care providers, online | Two eLearnings to increase knowledge on shared decision making were developed. |
| HeartGuard adults congenital heart disease | Digital interaction | Product |  |  |  |  |  |  |  |  | • |  | Cardiovascular | Monitoring | App, wearable | Patients and care providers, home-based | Home-based care by the use of mobile apps and a monitoring device for patients with congenital heart disease was developed and implemented as standard of care. |
| Design your own eHealth app | Digital interaction | Product |  |  |  |  |  |  |  |  | • | • | NA | Education, training | Website | Medical students, online | An online education module to develop your own medical app was developed. |
| ReValidate!-online! | Digital cohesiveness | Product |  | • |  |  |  |  | • |  | • |  | Musculoskeletal | Treatment | App, wearable | Patients, home-based | An online platform to support communication on rehabilitation's progress was developed. |
| *LUMC* |  |  | *2* | *3* | *4* | *5* | *6* | *7* | *8* | *9* | *10* | *11* | *Health domain* | *Health service* | *Technology%* | *Environment setting#* | *(Clinical) outcomes / evident benefits $* |
| Medical dashboard: Hospital data available for GP | Digital cohesiveness | Product |  |  | • | • |  | • | • |  |  |  | NA | Administration | EHR | General practitioner, general practice | An interconnection between hospital and general practice, to support exchanges of patient information was established. A legal framework was set. |
| PatientCoach: Patient profile chart* | Digital interaction | Product |  |  |  |  |  |  | • |  | • |  | Respiratory | Consultation, participation | Digital dashboard | Patients and physicians, hospital and general practice | A patient profile map was developed and piloted. Patients and physicians were positive about the profiling, but implementation was lacking behind because of personnel matters. |
| Changing mind and  metabolism | Digital interaction | Product |  |  |  |  |  |  | • |  | • |  | Diabetes | Education | App | Patients, home-based | A pre-diabetic app to track intake and provided feedback about food choices for patients at risk to develop diabetes, was developed and tested. |
| MyHeart Counts | Digital interaction | Product |  |  |  |  |  |  |  |  | • | • | Cardiovascular | Research | App | Researchers and physicians | Apple's ResearchKit app 'MyHartCounts' was translated to the Dutch market and an opensource toolkit for 'data collection with apps' was developed. |
| *Radboudumc* |  |  | *2* | *3* | *4* | *5* | *6* | *7* | *8* | *9* | *10* | *11* | *Health domain* | *Health service* | *Technology%* | *Environment setting#* | *(Clinical) outcomes / evident benefits $* |
| Experience and use of patient portals | Digital cohesiveness | Knowledge |  |  | • |  |  |  |  |  |  |  | NA | Process Information | Patient portal | NA | Knowledge was increased about implementation of patient portals. Important reason to implement a patient portal was the desire to share information with patients. |
| Reading physician's notes through the EMR | Digital interaction | Knowledge |  |  | • |  |  |  | • |  |  |  | NA | Process Information, administration | EHR, patient portal | Patients and physicians, home-based and hospital | Patients' and physicians' opinions concerning direct sharing of physicians notes through EHR and patient portal were analyzed. It may improve communication, but could mean more administration for the physician. |
| Sticking to your  exercises | Digital interaction | Product |  |  |  |  |  |  |  |  | • |  | Musculoskeletal | Treatment | App | Patients and physiotherapists, home-based | A personalized app to scan NFC stickers that present rehabilitations exercises was developed and tested. Self-efficacy was significantly improved by using the app. |
| eHealth working place for nurses | Digital interaction | Knowledge |  |  |  |  |  |  |  |  |  | • | NA | Education, research | NA | Nurses, digital environment | A qualitative study evaluated the barriers and facilitators of nurses who are digitally lagging. Active education and monitoring by the hospital were considered important to enhance digital literacy. |
| *VUMC* |  |  | *2* | *3* | *4* | *5* | *6* | *7* | *8* | *9* | *10* | *11* | *Health domain* | *Health service* | *Technology%* | *Environment setting#* | *(Clinical) outcomes / evident benefits$* |
| Patient portal for parents and children after IC admission | Digital cohesiveness | Product |  | • | • | • |  |  | • |  | • |  | Pediatrics | Information, administration | Patient portal, app | Parents and children, home-based | Content for a digital library, patient portal and app, in order to improve the information supply to parents of children who were admitted to the Intensive Care unit, was developed. Unfortunately the use of the patient portal was limited. |
| Patient portal use for medication reconciliation | Digital interaction | Knowledge |  |  |  |  | • |  | • |  | • | • | NA | Administration, participation | Patient portal | Patients and care providers, home-based | The use of a patient portal by patient to update their medication is low (27%). Both patients and care providers reckon the benefits, such as saving time and control of own health. |
| Interactive website Alzheimer center | Digital interaction | Product |  | • |  |  |  |  | • | • | • | • | Neurology | Education, consultation, participation | Website | Patients and informal caregivers, online | A forum and blog were developed and implemented on the Dutch Alzheimercentrum website. During development the patients suggested the modules should be clear simple and contain a manual. |
| Validation eHealth Impact Questionnaire-NL | Digital interaction | Product |  | • |  |  |  | • | • | • | • |  | NA | Research | NA | Researchers | The eHealth impact Questionnaire (eHIQ) was translated and validated for Dutch users. The eHIQ-NL underwent some changes of the subscales compared to the English version. |
| *UMCG* |  |  | *2* | *3* | *4* | *5* | *6* | *7* | *8* | *9* | *10* | *11* | *Health domain* | *Health service* | *Technology%* | *Environment setting#* | *(Clinical) outcomes / evident benefits$* |
| eHealth literacy | Digital interaction | Knowledge |  |  |  |  |  |  | • |  | • | • | Chronic renal diseases | Research | Patient portal | Researchers | EHealth literacy skills of patients with chronic kidney disease were investigated and a prototype of a patient portal for this group of patients with low literacy skills was developed. Simple design and easy navigation were considered important. |
| eHealth partnership | Digital interaction | Knowledge |  |  |  |  |  | • | • | • |  |  | NA | Research | NA | Researchers | Quality criteria and requirements in order to enhance the use of eHealth systems by elderly (age 75+) were investigated. The systems have to serve a clear goals and be very user-friendly. Communication through the patient portal is considered of added value by care providers. |
| Evaluation of patient portal for elderly | Digital cohesiveness | Knowledge |  |  | • | • | • |  |  | • |  |  | NA | Research | Patient portal | Researchers | A qualitative study was conducted to evaluate the use of an online health record by elderly (age 50-85). Use of navigation can be difficult. When developing a patient portal, create clear home and return buttons and take disabilities of vision-impaired people into account. |
| *MUMC* |  |  | *2* | *3* | *4* | *5* | *6* | *7* | *8* | *9* | *10* | *11* | *Health domain* | *Health service* | *Technology%* | *Environment setting#* | *(Clinical) outcomes / evident benefits$* |
| Monitoring Parkinson's Disease at home | Digital interaction | Product |  | • |  |  |  |  |  |  | • |  | NA | Monitoring, diagnostics | Wearable | Patients and physicians, home-based | A wearable that measures Parkinson's symptoms in a home setting was developed and experiences of 20 patients were collected. Patients were confident wearing the wearable and half of the patient are willing to wear it longer than two weeks. |
| Fundamentals of Clinical Data Science (eBook) | Digital interaction | Product |  | • |  |  |  |  |  |  | • | • | NA | Education | eBook | Care providers | An open access eBook was composed to increase basic knowledge of clinical data science (e.g. machine learning, predictive analytics). |
| My kneesurgery* | Digital interaction | Product |  |  |  |  |  |  | • |  |  |  | Musculoskeletal | Consultation, participation | App | Patients and care providers, hospital | An app that supports composing an individual care pathway when undergoing knee surgery, was developed. Planned implementation and testing was not possible yet due to resistance to change by physicians. |
| *Erasmus* |  |  | *2* | *3* | *4* | *5* | *6* | *7* | *8* | *9* | *10* | *11* | *Health domain* | *Health service* | *Technology%* | *Environment setting#* | *(Clinical) outcomes / evident benefits$* |
| Blue button to Patient Portal | Digital cohesiveness | Product |  |  | • | • | • | • | • | • | • |  | NA | Administration | Patient portal | Patients, home-based | The project effectuated the addition of a 'Blue Button' to the patient portal, through which patients can download their medical information in XML format. |
| Consult replay* | Digital interaction | Product | • |  | • |  |  | • | • |  | • | • | NA | Administration, information | Audio recording, EHR, patient portal | Patients and care providers, home-based and hospital | The project effectuated the possibility to replay a conversation between patient and care giver, by recording the audio of a conversation through the EHR and uploading it to the patient portal. A legal framework was developed. Testing was not yet performed due to a lack of interoperability of systems. |
| Value Based Health Care (PROMIS)* | Digital interaction | Product |  |  |  | • | • |  | • |  |  |  | NA | Administration, research | EHR | Researchers | The project aimed to implement the PROMIS method. However, due to an IT-freeze in the executing hospital, this project showed unsatisfactory progress at the mid-term evaluation and was therefore terminated prematurely. |
| *UMCU* |  |  | *2* | *3* | *4* | *5* | *6* | *7* | *8* | *9* | *10* | *11* | *Health domain* | *Health service* | *Technology%* | *Environment setting#* | *(Clinical) outcomes / evident benefits$* |
| Wireless Vitals | Digital cohesiveness | Product |  |  | • |  | • |  | • |  | • |  | Cancer | Monitoring, diagnostics | Wearable | Patients and care providers, home-based and hospital | Four wireless wearables, to monitor post-operative patients in home setting, were evaluated on accuracy and 'unintended consequences' of telemonitoring. Three of the four wearable were accurate, however a ‘nurse's worry' was considered important as well. |
| PROfeel: Rendering insight in fatigue | Digital interaction | Product |  |  |  |  |  |  | • |  | • | • | Pediatrics | diagnostics, consultation | App | Patients and care providers, home-based | A new method to conduct a personalized 'Ecological Momentary Assessment' (EMA) by children with chronic fatigue complaints, was developed. The EMA was included in an app and feasibility of conducting the EMA through an app was approved. |
| SAFE@HOME | Digital interaction | Product |  |  |  |  |  |  | • | • | • |  | Obstetrics and gynecology | Diagnostics, monitoring | App, wearable | Patients and care providers, home-based and hospital | An app connected with a wireless blood pressure meter and care strategy to telemonitor the tension of high risk pregnant women, was developed. Pregnant women were highly satisfied about the digital solution. |
| Get-it | Digital interaction | Product |  |  |  |  |  | • | • |  | • |  | Mental | Information, participation | App | Patients, home-based | An app was developed together with patients to support patients with memory problems. The use of the app was evaluated and patients experience better perception of their memory loss. |
| *UMC: University Medical Center; AMC: Academic Medical Center; LUMC: Leiden University Medical Center; Radboudumc: Radboud University Medical Center; VUMC: Vu Medical Center; UMCG: University Medical Center Groningen; MUMC: Maastricht University Medical Center; Erasmus MC: Erasmus Medical Center; UMCU: University Medical Center Utrecht.  &See textbox 1 for description of deliverables, note deliverable 1 is lacking because deliverable 1 comprised an individual assignment; %Technology describing (if applicable): the studied or developed eHealth solution; #environment setting describing (if applicable): people and locations; $(Clinical) outcomes and evident benefits belonging to the Socio-economic analysis aspect of the framework. | | | | | | | | | | | | | | | | | |
