# Supplementary material for: Successes of and Lessons From the First Joint eHealth Program of the Dutch University Hospitals: Evaluation Study
Source: J Med Internet Res. 2021 Nov 25;23(11):e25170. doi: 10.2196/25170 (PMC8663485; doi:10.2196/25170)
Supplement: Multimedia Appendix 4 [file jmir_v23i11e25170_app4.docx]

Multimedia Appendix 4 – Interview quotes

**Theme: success factors for eHealth development and implementation**

**Fulfill a need**

- *‘We did not carry out an official need assessment, but there was a clear problem for which we were looking for a solution. It's not that we thought ‘hey’ we have technology let’s see how reliable that is, but we have a problem let’s solve that’* (Project leader 1);
- *'A need from healthcare provider and patient is important, what is the problem that I want to solve and is it a problem at all?’* (Project leader 4);
- ‘*Steve Jobs said ‘I don't do market research’ and quoted Henry Ford: ‘If I had asked my customers what they would like, they would have said a faster horse’, but he came up with a car’* (Project leader 6).

**Outsource**

- *‘Nice to have a commercial partner. Industry that has the right people. Very simple things such as offering a service to deliver a device. And, it is so beautiful that the commercial party, for example, has taken on the service of explaining apps and that we were able to focus on healthcare’* (Project leader 8).

**Communication**

- *‘Miscommunication between researchers and IT-developers is a theme itself’* (Project leader 7);
- *‘We had ideas in advance and during the course of the project those ideas change a bit. During the conversations with IT-developers, they would interpret information in a certain slightly different way. Now I would put much more down on paper to be clearer’* (Project leader 3).

**Personnel**

- ‘*That a project is someone's ‘pet project’ and the project leader is really committed to it’* (Project leader 3)
- *‘Time to work on the project had to come almost fully from my own spare time, and also just working very hard’* (Project leader 4)
- *‘Yes I have seen that with other projects too, it can get out of hand if you depend on external parties. On the other hand, it also has everything to do with project leadership’* (Project leader 5)*.*

**Theme: essential third parties**

**IT-services**

- *‘Most hospitals spent a very small percentage of their budget on IT, they should really increase the budget in order to make other eHealth projects and IT solutions integrate much easier’* (Project leader 1);
- *‘That comes down to sufficient capacity of IT-services*’ (Project leader 2);
- *‘We cannot build an eHealth solution and you really need to have an IT person that can listen to the needs of a customer, otherwise it is not workable’* (Project leader 7).

**Medical Ethical Committee**

- *‘Ethical approval took half a year, causing project delay due to an ethical discussion. Technologies and possibilities are really completely new for the ethical commission. It works well to inform them thoroughly and to show the impact of this type of technology’* (Project leader 1).

**Legal**

- ‘*The New General Data Protection Regulation in the EU must ensure that it is all safer, but actually that GDPR has ensured that people are put on the brakes even more because people are not entirely sure what is and what is not possible’* (Project leader 7);
- ‘*The data goes to a cloud server in The United States of America, so at this moment I had to write a data management plan and write a PIA. And there still must be an agreement arranged with that American company. That takes quite some time’* (Project leader 1).

**Theme: flexibility**

**Project planning**

- *Do what you, as a project leader, have promised and stick to the planning. If the planning changes, make adjustments timely’* (Project leader 5);
- ‘*A planning is good to have with concrete these steps knowing what must be done and when, but in the course of the project you find out that things can also be arranged in another order or at a different time’* (Project leader 3);
- ‘*In principle, planning is important and it is good to have a final goal. Perhaps it is handy, future wise, to be a bit more flexible in this type of calamity and in such cases, for example, add a month to it or something’* (Project leader 7).

**Conducting research**

- *‘Patient participation to research is not easily organized, it is always underestimated. That took a lot of effort, also to get focus groups together, try luring 6-7 patients with a sandwich to your hospital in the evening’* (Project leader 5);
- ‘*Everyone actually says yes to participation, but of course it is also relatively low burden. These patients really have a problem and if you explain what the intention is then they will see the added value themselves*’ (Project leader 6);
- *‘That it is fairly new and innovative, so no literature. End points were difficult to determine. Afterwards sometimes we thought whether we made the right choices’* (Project leader 8).

**Effectiveness testing**

- *‘I think it is always good to study eHealth projects in a study context, but I think it should not be an obstacle to the development of an eHealth solution, so you will have to test in very short cycles with small questions in a small group. It does not have to be that a certain big RCT is needed to get the evidence’* (Project leader 1);
- *‘With a lot of eHealth solutions and apps, you only have data about the first 2-3 months of use, which overestimate the effect. Research to investigate long-term effects is a very important missing factor’* (Project leader 4);
- ‘*You must have your CE-marking, but it does not tell everything, it certainly does not say anything about effectiveness’* (Project leader 5);
- ‘*You must be sure that the care delivered by the eHealth solution is not of poorer quality than the care you normally provide’* (Project leader 7).
